# Educational delays and psychological burden by disability status among newly graduated medical students in Japan

**DOI:** 10.1002/pcn5.70287

**Published:** 2026-01-18

**Authors:** Kana Kiryu, Hidetaka Tamune, Hirohisa Fujikawa, Chihiro Kakiuchi, Hiroyuki Harada, Masanobu Ito, Takashi Watari, Yuji Nishizaki, Tadafumi Kato, Yasuharu Tokuda

**Affiliations:** ^1^ Department of Psychiatry and Behavioral Science Juntendo University Graduate School of Medicine Tokyo Japan; ^2^ Department of General Medicine Juntendo University Faculty of Medicine Tokyo Japan; ^3^ Integrated Clinical Education Center, Kyoto University Hospital Kyoto Japan; ^4^ Division of Medical Education Juntendo University School of Medicine Tokyo Japan; ^5^ Muribushi Okinawa for Teaching Hospitals Urasoe Okinawa Japan; ^6^ Tokyo Foundation for Policy Research Tokyo Japan

The prevalence of people with disabilities has been increasing in medical education and healthcare settings worldwide.^1^ Previous research has demonstrated that job accommodations decreased the risk of depression and self‐reported medical errors.^2^ Accordingly, it is important to consider educational and workplace accommodations not only to enhance the welfare of individuals with disabilities but also to improve workplace safety and functioning. However, educational and workplace accommodations remain unexamined in Japan. This study aimed to obtain foundational data on disability among newly graduated medical students by assessing access to accommodations and examining the differences in academic delays and psychological burdens associated with disabilities.

We surveyed postgraduate year (PGY)−0 residents at the very start of their residency training as they completed the General Medicine In‐Training Examination (GM‐ITE) from April to May 2025. The GM‐ITE is a nationwide computer‐based examination taken by approximately half of Japan's residents and is validated for assessing clinical knowledge.^3^, ^4^ After completing the GM‐ITE, candidates were invited to participate in this study. Of the 748 eligible examinees, 281 provided written informed consent and were enrolled. Subsequently, 44 were excluded for missing psychological assessment data and 2 for selecting “Prefer not to answer” to disability status, yielding a final sample of 235 participants (31.4%; Figure S1).

We assessed health‐related quality of life using the EuroQol 5 Dimensions 5‐Level (EQ‐5D‐5L) scale.^5^ Psychological distress was measured with the Kessler 6‐Item Psychological Distress Scale (K6).^6^ Work engagement was evaluated using the Utrecht Work Engagement Scale (UWES).^7^

The variables were summarized and analyzed according to their distribution. Categorical variables with small expected cell counts were compared using Fisher's exact test. Continuous variables with non‐normal distributions were analyzed using the Wilcoxon rank‐sum test, and group differences were reported as median differences with Cliff's delta. GM‐ITE total scores, as continuous variables with approximately normal distributions, were analyzed using Welch's *t*‐test, and group differences were reported as mean differences with Hedges' *g*. Statistical analyses were performed using R software, version 4.4.1.

Table 1 summarizes the characteristics of the participants, academic delays, psychological assessment scores, and GM‐ITE scores by group. A total of 13 participants (5.5%) responded “yes” to the disability status question, while the remaining 222 participants responded “no.” Among these 13 individuals, 2 (15.4%) had access to reasonable accommodation. Characteristics of the group with disabilities are shown in Table S1. Due to the small sample size, we did not perform comparative analyses between those with and without accommodations, nor did we conduct subgroup analyses by disability type.

**Table 1:** Characteristics of the participants (*N* = 235).

	All (235)	With disability (13)	Without disability (222)	Difference (95% CI)	Effect size (95% CI)	*p*‐Value
Age (median, Q1–Q3)^†^	25.0 (24.0, 26.0)	25.0 (24.0, 27.0)	25.0 (24.0, 26.0)	‐	‐	0.47
Sex, *n* (%)^‡^						0.38
Male	151 (64.3%)	10 (76.9%)	141 (63.5%)	‐	‐	
Female	76 (32.3%)	2 (15.4%)	74 (33.3%)	‐	‐	
Prefer not to answer	8 (3.4%)	1 (7.7%)	7 (3.2%)	‐	‐	
Type of hospital, *n* (%)^‡^						0.65
Community hospital	163 (69.4%)	8 (61.5%)	155 (69.8%)	‐	‐	
University main hospital	67 (28.5%)	5 (38.5%)	62 (27.9%)	‐	‐	
University branch hospital	5 (2.1%)	0 (0%)	5 (2.3%)	‐	‐	
Location of hospital, *n* (%)^‡^						0.55
Rural	154 (65.5%)	10 (76.9%)	144 (64.9%)	‐	‐	
Urban	81 (34.5%)	3 (23.1%)	78 (35.1%)	‐	‐	
Hospital size, *n* (%)^†^						0.22
<400 beds	41 (17.4%)	4 (30.8%)	37 (16.7%)	‐	‐	
400–599 beds	87 (37.0%)	4 (30.8%)	83 (37.4%)	‐	‐	
600–799 beds	38 (17.1%)	0 (0%)	38 (17.1%)	‐	‐	
≥800 beds	69 (29.4%)	5 (38.5%)	64 (28.8%)	‐	‐	
Use of accommodation, *n* (%)	N.A.	2 (15.4%)	N.A.	‐	‐	‐
Number of university absence days per year^†^	0.0 (0.0, 0.0)	0.5 (0.0, 4.8)	0.0 (0.0, 0.0)	0.5 (0.0, 8.0)^¶^	0.5 (0.1, 0.8)^‡‡^	<0.001***
Number of hospital visit days per year^†^	0.0 (0.0, 0.0)	5.5 (2.0, 10.5)	0.0 (0.0, 0.0)	5.5 (0.5, 12.0)^¶^	0.8 (0.5, 1.0)^‡‡^	<0.001***
Years of delayed progression^†^	0.0 (0.0, 0.0)	0.0 (0.0, 1.0)	0.0 (0.0, 0.0)	0.0 (0.0, 1.0)^¶^	0.2 (0.0, 0.5)^‡‡^	0.0068**
EQ‐5D‐5L^†^	1.00 (0.87, 1.00)	0.90 (0.77, 1.00)	1.00 (0.87, 1.00)	−0.10 (−0.23, 0.00)^¶^	−0.22 (−0.53, 0.10)^‡‡^	0.14
K6^†^	1.0 (0.0, 3.0)	1.0 (1.0, 4.0)	1.0 (0.0, 3.0)	0.0 (0.0, 3.0)^¶^	0.2 (−0.1, 0.5)^‡‡^	0.15
UWES^†^	3.3 (3.0, 4.2)	3.7 (3.0, 4.3)	3.3 (3.0, 4.0)	0.3 (−0.3, 1.0)^¶^	0.1 (−0.2, 0.4)^‡‡^	0.57
GM‐ITE total score^§^	42.7 (6.8)	41.8 (7.3)	42.7(6.8)	−0.9 (−5.4, 3.6)^††^	−0.1 (−0.7, 0.4)^§§^	0.66

*Note*: The EQ‐5D‐5L index was calculated using the Japanese value set derived from the composite time trade‐off method and represents the Quality‐Adjusted Life Year score on a ratio scale, where 0 indicates death, and 1 indicates full health. The K6 is a psychological distress scale developed to screen for mental health conditions, such as depression and anxiety. Total scores ranged from 0 to 24, with higher scores indicating greater psychological distress. The Japanese version of the UWES is a self‐reported instrument designed to assess work engagement. The scores range from 0 to 6, with higher scores indicating greater work engagement. The GM‐ITE consists of four domains: medical interviews and professionalism, symptomatology and clinical reasoning, physical examination and clinical procedures, and clinical diseases. It comprehensively covers a wide range of medical fields, including internal medicine, surgery, pediatrics, obstetrics, gynecology, and psychiatry. The test is administered in a computer‐based format, with total scores ranging from 0 to 60. Statistical methods, according to their distribution, are as follows: †, Wilcoxon test; ‡, Fisher's exact test; §, Welch's test; ¶, median difference; ††, mean difference; ‡‡, Cliff's *d*; §§, Hedge's *g*.

Abbreviations: 95% CI, 95% confidence interval; EQ‐5D‐5L, EuroQol 5 Dimensions 5‐Level; GM‐ITE, General Medicine In‐Training Examination; K6, Kessler 6‐Item Psychological Distress Scale; N.A., not assessed; UWES, Utrecht Work Engagement Scale.

**p < 0.01, and ***p < 0.001.

In this study, only a minority of newly graduated medical students with disabilities had access to reasonable accommodations. Prior US research reported that 11.9% of PGY‐0 residents identified as having a disability, 48.0% of whom required accommodations, and 49.4% of those had requested them.^8^ Both the United States^8^ and the present study in Japan indicate that fewer than one‐quarter of disability‐identified individuals received accommodations. Given the benefits of accommodations, more attention should be paid to individuals with disabilities who wish to receive accommodations but currently lack access.

Differences in educational delays were observed between groups; however, no significant differences were identified in psychological burden or GM‐ITE total scores between participants with and without disabilities. A previous study showed a gap between the number of people who identify as having a disability among medical students (14%) and physicians in academic centers (5%), indicating attrition along the pathway.^9^ Another study demonstrated that mental ill‐health symptoms contribute to students' intentions to discontinue their medical education.^10^ Our study may reflect a similar phenomenon, in which the highly selective process of university entrance through the National Medical Licensing Examination functions as a survivorship bias whereby students with disabilities who experience substantial psychological burdens may discontinue their medical training at earlier stages. Burdens that should be mitigated through accommodation may be placed on individual effort and personal sacrifice. Limited access to accommodation may restrict career options and hinder efforts to promote diversity among healthcare professions.

This study had limitations. First, the cross‐sectional design prevented causal inferences regarding the relationship between disability identity and outcomes. Second, only 13 participants self‐reported having a disability, possibly reflecting barriers to disclosure. To minimize potential self‐selection bias, the survey was conducted anonymously. Nonetheless, these data provide information on the current status of disability identity and access to accommodation among PGY‐0 physicians.

To our knowledge, this study is the first to investigate disabilities and accommodations among newly graduated medical students in Japan. We found that individuals with disabilities experienced greater academic delays, while access to accommodations was limited. Additional research with larger and more diverse samples, including detailed information on accommodation use, is required to inform future efforts towards creating more inclusive environments.

## AUTHOR CONTRIBUTIONS

Hidetaka Tamune, Hirohisa Fujikawa, and Yuji Nishizaki conceived the study with input from Kana Kiryu, Chihiro Kakiuchi, Hiroyuki Harada, Masanobu Ito, Tadafumi Kato, and Yasuharu Tokuda. Kana Kiryu analyzed the data and drafted the first manuscript, supervised by Hidetaka Tamune. All authors discussed, proofread, and approved the final manuscript.

## CONFLICT OF INTEREST STATEMENT

H.T. has received an honorarium from JAMEP for preparing the GM‐ITE exam. T.W. has received an honorarium from JAMEP as a lecturer at seminars and training sessions organized by JAMEP. Y.N. has received an honorarium from JAMEP as the GM‐ITE project manager. Y.T., the director of JAMEP, has received an honorarium from JAMEP as a speaker at JAMEP lectures. T.K. is an Editorial Board member of *Psychiatry and Clinical Neurosciences Reports* and a co‐author of this article. To minimize bias, he was excluded from all editorial decision‐making. The remaining authors declare no conflict of interest.

## ETHICS APPROVAL STATEMENT

This study was approved by the Ethics Review Board of JAMEP (Approval No. 24‐24).

## PATIENT CONSENT STATEMENT

All participants voluntarily provided informed consent through an on‐screen prompt.

## CLINICAL TRIAL REGISTRATION

N/A.

## Supporting information

Supporting Information.

## Data Availability

The data that support the findings of this study are available on request from the corresponding author. The data are not publicly available due to privacy or ethical restrictions.
