# Supplementary material for: Educational delays and psychological burden by disability status among newly graduated medical students in Japan
Source: PCN Rep. 2026 Jan 18;5(1):e70287. doi: 10.1002/pcn5.70287 (PMC12812281; doi:10.1002/pcn5.70287)
Supplement: Supplementary file 1 — Supporting Information. [file PCN5-5-e70287-s001.docx]

**Educational Delays and Psychological Burden by Disability Status Among Newly Graduated Medical Students in Japan**

Kana Kiryu MD^1^, Hidetaka Tamune MD, PhD^1*^, Hirohisa Fujikawa MD, PhD^2^, Chihiro Kakiuchi, MD, PhD^1^, Hiroyuki Harada, MD, PhD^1^, Masanobu Ito MD, PhD^1^, Takashi Watari MCTM, DTMH, MHQS, MD, PhD^3^, Yuji Nishizaki MD, MPH, PhD^4^, Tadafumi Kato MD, PhD^1^, Yasuharu Tokuda MD, MPH^5,6^

**SUPPLEMENTARY MATERIALS**

**Fig. S1** Flowchart of participant selection


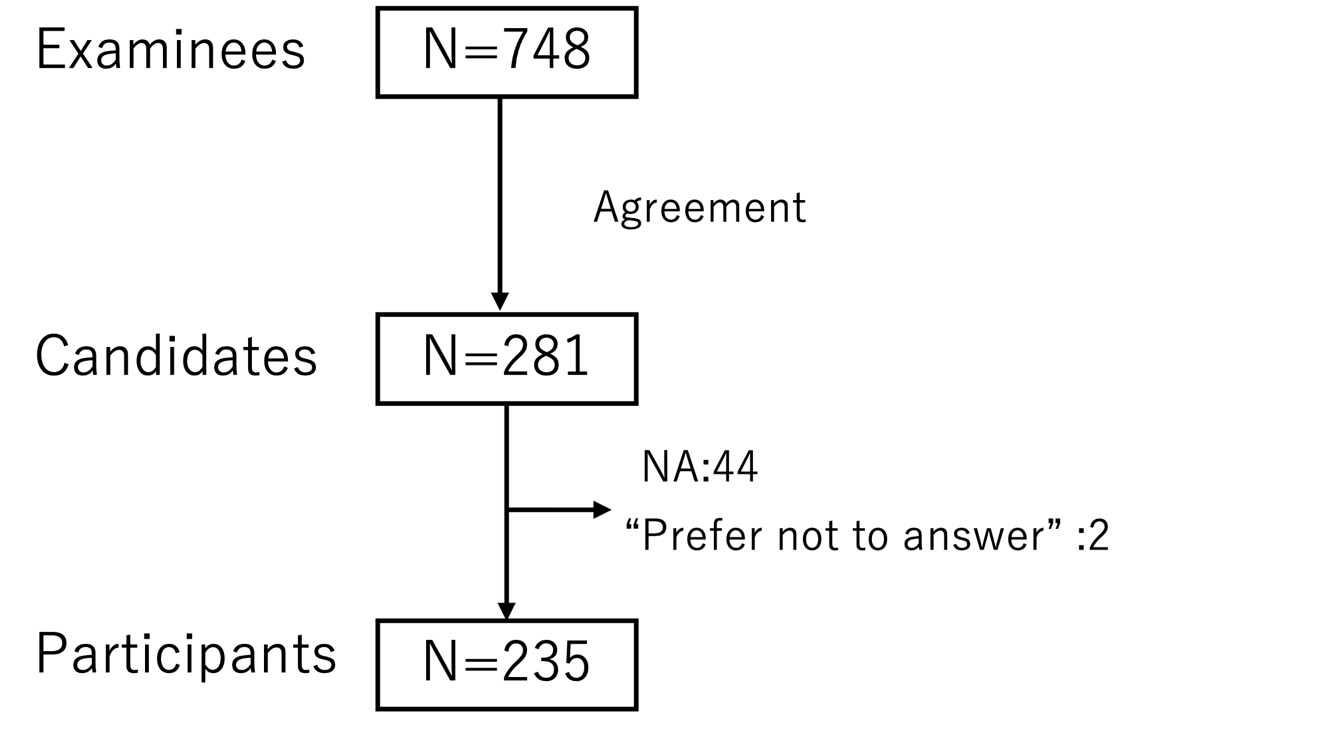


**Fig. S1** Flowchart of participant selection

Participants were recruited from General Medicine In-Training Examination (GM‐ITE) examinees in 2025. Candidates received an explanation of the study and those who provided consent were included as participants. Two participant was excluded because they selected “Prefer not to answer” to the disability status question. NA = not assessed.

**Fig S2.** Box-and-whisker plots of academic delays, psychological assessments, and GM-ITE total scores


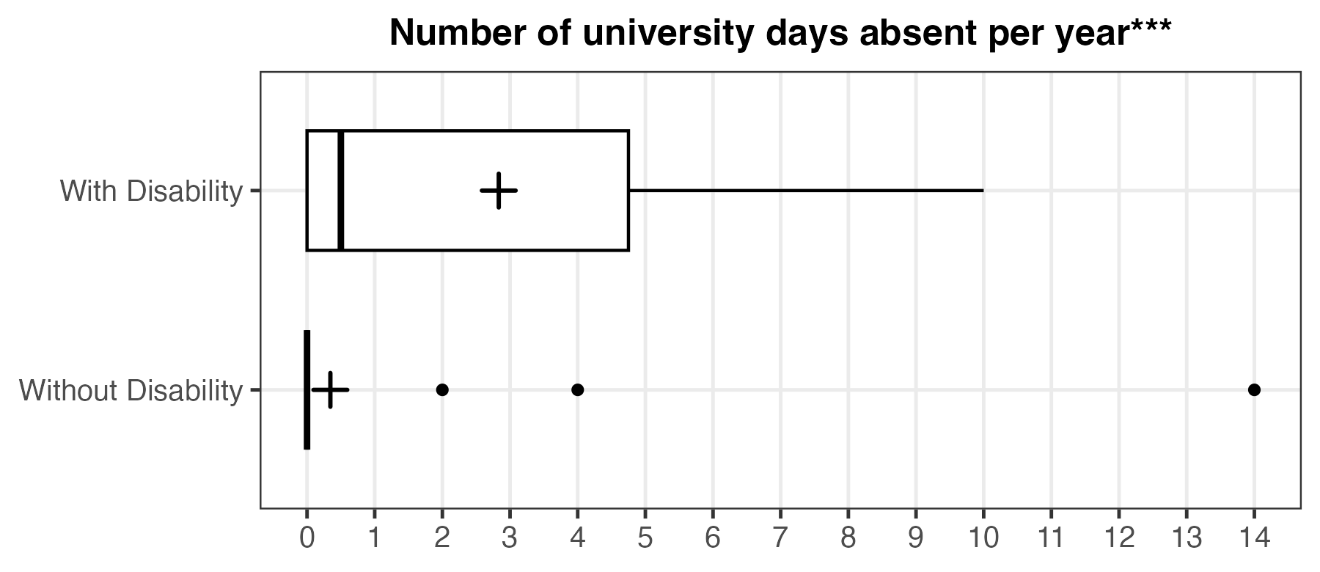


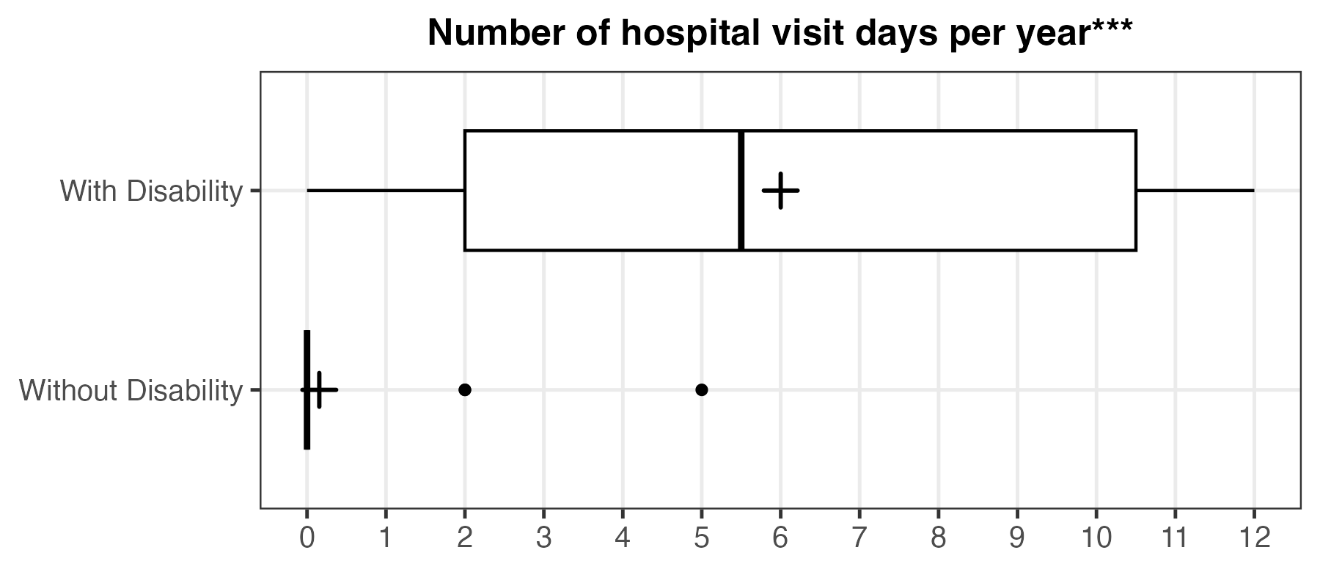


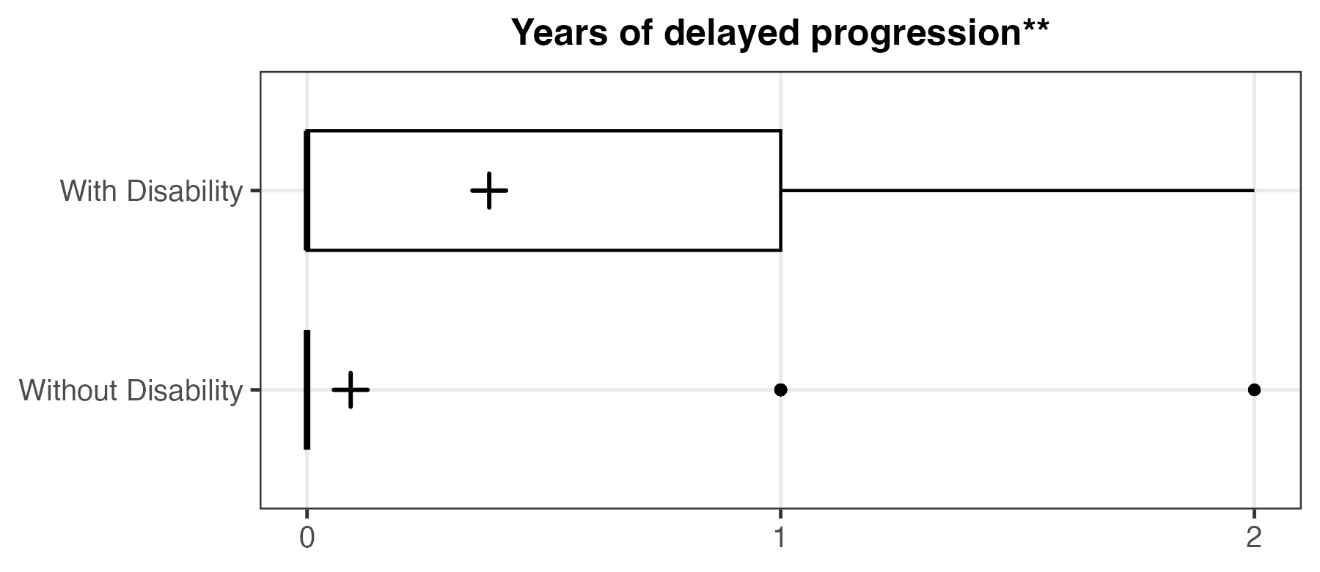

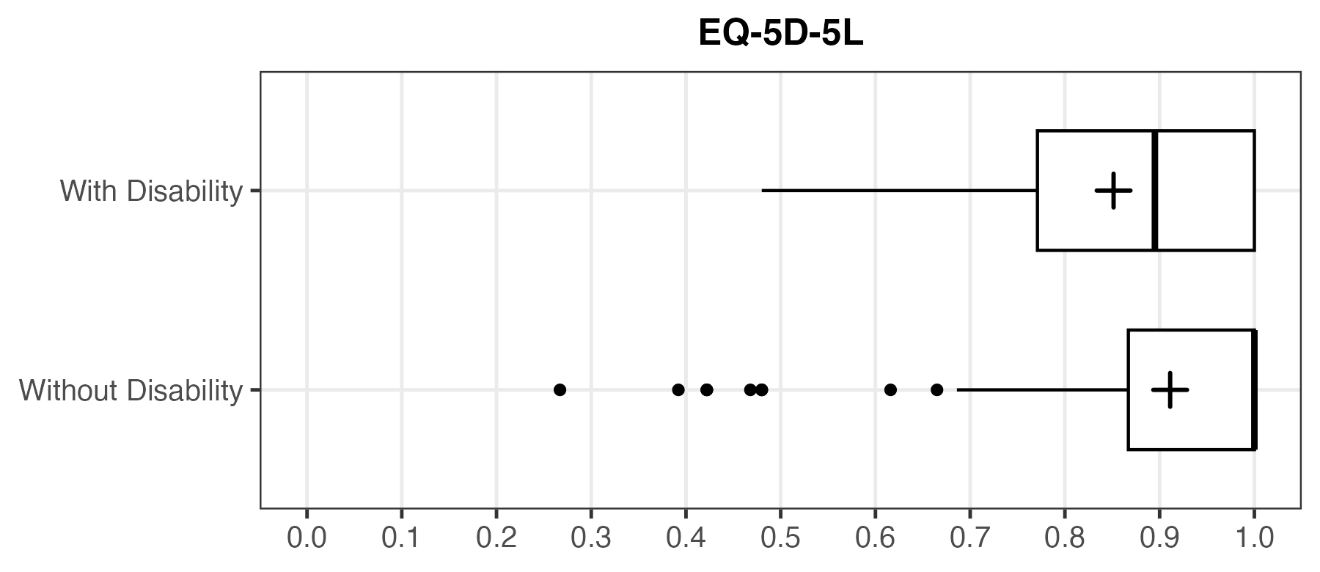


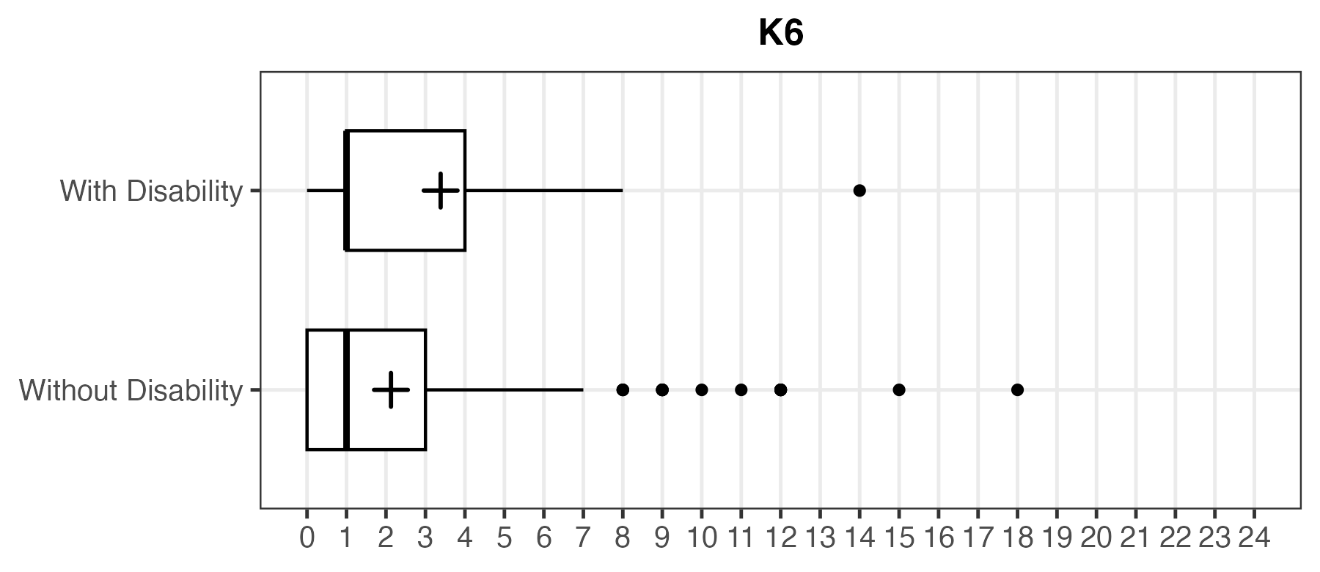

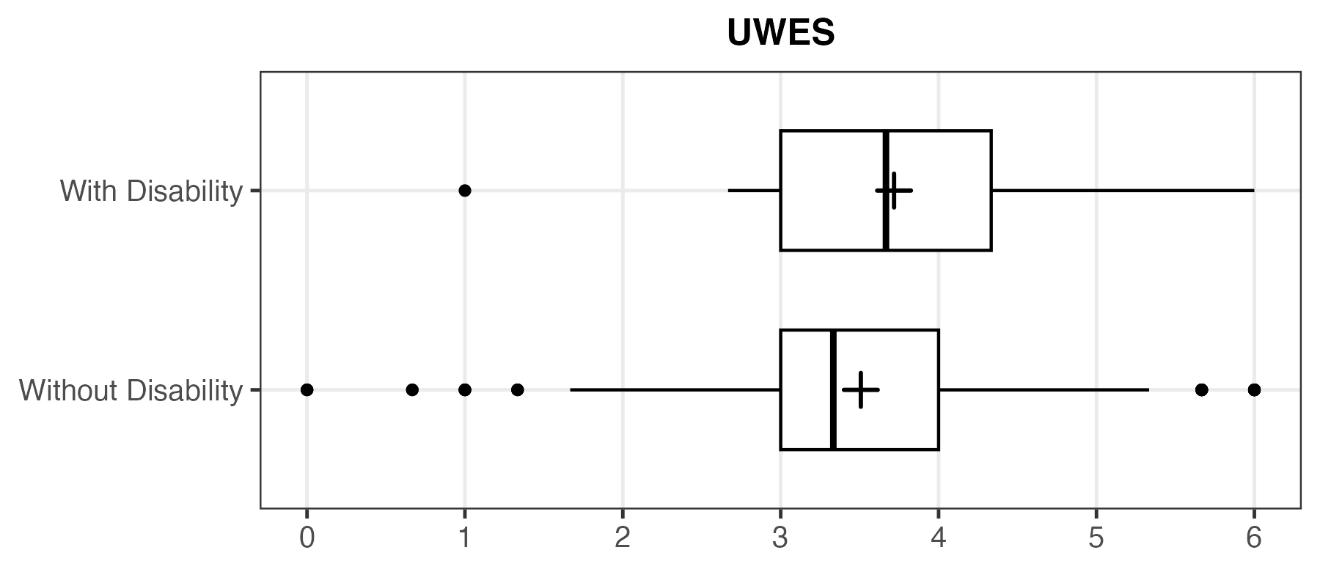

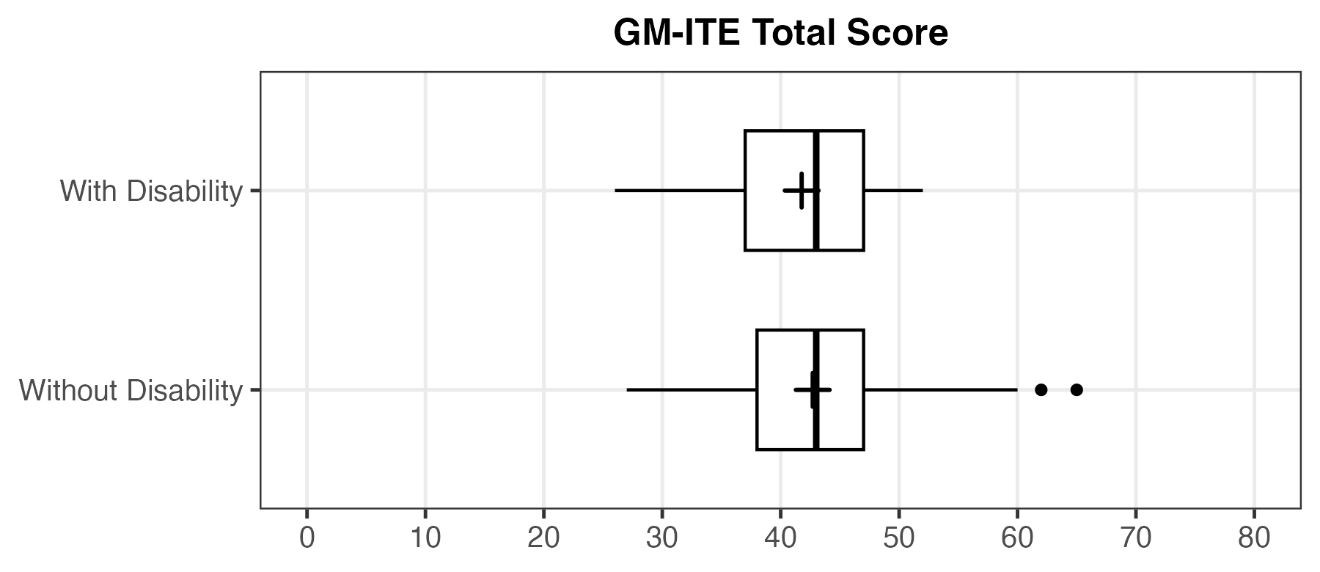


**Fig S2.** Box-and-whisker plots of academic delays, psychological assessments, and GM-ITE total scores

Box-and-whisker plots comparing academic delays (number of university absences, number of hospital visits, and years of delayed progression), psychological assessment scores (EQ-5D-5L, K6, and UWES), and total GM-ITE scores. Mean scores are represented by “+” signs and median scores by vertical lines within the boxes. The widths of the boxes represent interquartile ranges. Tukey’s method was used to calculate whiskers and outliers (represented by solid black dots). Outliers were identified for observational purposes and were therefore included in all analyses. GM-ITE: General Medicine In-Training Examination; EQ-5D-5L; EuroQol 5 Dimensions 5-Level scale; K6: Kessler 6-Item Psychological Distress Scale; UWES: Utrecht Work Engagement scale* *p* < 0.05, ** *p* < 0.01, *** *p* < 0.001.

**Table S1.** Characteristics of the group with disability (N = 13 individuals)

Participants who self-identified as having a disability indicated their type of disability using multiple-choice questions. The response categories are listed. One participant selected both “developmental disorder” and “mental disorder,” so the total does not equal 13.

| **Types of disabilities** |  |
| --- | --- |
| Visual impairment | 2 |
| Hearing impairment | 0 |
| Physical disability | 1 |
| Chronic medical condition or physical illness | 2 |
| Intellectual disability | 0 |
| Developmental disorder | 3 |
| Mental disorder | 3 |
| Other disability | 3 |
